# Urinary bisphenol A concentrations and the risk of obesity in Korean adults

**DOI:** 10.1038/s41598-021-80980-8

**Published:** 2021-01-15

**Authors:** Shinje Moon, Moon Young Seo, Kyungho Choi, Yoon-seok Chang, Shin-Hye Kim, Mi Jung Park

**Affiliations:** 1 Department of Internal Medicine, Kangnam Sacred Heart Hospital, Hallym University Medical Center, Hallym University College of Medicine, Seoul, 07441 Republic of Korea; 2 Department of Pediatrics, Sanggye Paik Hospital, Inje University College of Medicine, 1342, Dongilro, Nowon-gu, Seoul, 01757 Korea; 3 Department of Environmental Health Sciences, Seoul National University, Seoul, 08826 Republic of Korea; 4 Division of Environmental Science and Engineering, Pohang University of Science and Technology (Postech), Pohang, 37673 Republic of Korea

**Keywords:** Obesity, Environmental impact

## Abstract

This study was aimed to evaluate the association between urinary bisphenol A (BPA) levels and risk of obesity in Korean adults. We analyzed data from the Korean National Environmental Health Survey (KoNEHS) Cycle 2 (2012–2014) and Cycle 3 (2015–2017). A total of 10,021 participants aged ≥ 19 years were included. Urine dilution was corrected by the covariate-adjusted standardization (CAS) method. We performed meta-analysis, logistic regression analysis by matching all covariates with a 1:1 propensity score, and a 4-knot restricted cubic spline plot model to calculate the odds ratios (ORs) for obesity according to natural log-transformed BPA levels. Mean urinary BPA concentration was 1.12 µg/L in KoNEHS Cycle 2 and 1.32 µg/L in Cycle 3. BPA levels were significantly higher among obese adults than among non-obese adults in both KoNEHS Cycles 2 and 3. In pooled data of KoNEHS Cycles 2 and 3, BPA showed significant positive associations with ORs for obesity in both sexes, which were more prominent in females (linear) than in males (non-linear). These associations were confirmed in spline analyses. CAS-applied BPA concentrations were positively associated with obesity in nationwide representative samples of Korean adults. Further studies are warranted to confirm and elucidate the underlying mechanism.

## Introduction

Obesity constitutes a global epidemic, with a worldwide prevalence of overweight or obesity of 33%^1^. While excessive energy intake and sedentary lifestyles are known contributors, there has been increasing evidence to show that environmental chemicals may contribute to the development of obesity^2^. Among these environmental chemicals, bisphenol A (BPA) has been extensively investigated^3,4^. Because of its resemblance to estrogen in terms of molecular structure, BPA can interfere with estrogen action through nuclear receptors^5^. Previous studies indicated that BPA exposure might predispose animals and humans to obesity by inducing adipogenesis and lipogenesis^6,7^, modulating adipokine secretion, epigenetic modifications^8^, and alteration of thyroid function^9^.

Although well-designed prospective studies on the link between BPA exposure and obesity risk have been limited thus far, large-scale cross-sectional studies from the US, China, and Canada have generally suggested positive associations between urinary BPA and obesity risk in adults^10–13^, while reports suggesting otherwise are also available^14^. Meanwhile, among Korean adults, only two studies have investigated the relationship between BPA exposure and the risk of obesity, and their findings were inconsistent. The first study based on the Korean National Human Biomonitoring Survey 2009 (n = 1,870) reported no significant associations between urinary BPA and obesity status^15^. The most recent study using the Korean National Environmental Health Survey (KoNHES) Cycle 2 (n = 6,123) showed that urinary BPA levels were positively associated with obesity risk only in females, but not in males^16^.

Notably, most of the above-mentioned studies have been conducted after adjusting for urinary creatinine concentrations as independent covariates to account for urinary dilution of the BPA concentration. Controlling for urinary creatinine levels have been widely used in epidemiologic studies investigating the health effect of toxic chemicals based on their urinary concentrations. However, urinary creatinine levels could be influenced not only by the urinary dilution but also by age, sex, and muscle mass^17^. Since obesity is generally assessed by body mass index (BMI), which is associated with the muscle mass of a given individual, the amount of urinary creatinine excretion might be influenced by obesity status. Therefore using urinary creatinine as a separate covariate, especially in obesity research could induce collider stratification bias^18^. To overcome this limitation, a novel method, called covariate-adjusted standardization (CAS), has been recently introduced^19,20^.

In this study, we aimed to evaluate the association between CAS-applied urinary BPA concentrations and the risk of obesity, using pooled data from the KoNEHS Cycle 2 (2012–2014) and Cycle 3 (2015–2017).

## Materials and methods

### Study population

The KoNEHS is a cross-sectional, nationwide representative survey, which has been performed every 3 years since 2009 by the National Institute of Environmental Research, South Korea (NIER). The purpose of the KoNEHS was to provide baseline information on environmental chemical exposure to guide environmental health policy based on a legal provision (Environmental Health Act). KoNEHS was designed to represent the entire population. For regional allocation, the method taking proportional to the square root of the population size was applied. The KoNEHS collects data on socioeconomic status, health conditions, questionnaire-based personal interviews on environmental chemical exposure, physical examinations, and laboratory tests. More detailed information on the study design and methods is described in previous studies^21–23^. Written informed consent was obtained from all participants in KoNEHS. The KoNEHS dataset is de-identified and publicly available.

We included participants aged ≥ 19 years from the KoNEHS Cycle 2 (2012–2014) and Cycle 3 (2015–2017). After excluding 244 participants (220 from Cycle 2 and 24 from Cycle 3) owing to missing data on socioeconomic status, anthropometric features, urinary BPA concentrations, and creatinine levels, 6,258 participants from KoNEHS Cycle 2 and 3,763 participants from KoNEHS Cycle 3 were included (Supplementary Fig. 1 online).

### Measurement of bisphenol A

Random spot urine samples were collected and stored for less than 24 h following the standard procedures established by the NIER^24,25^. All institutions engaged with the chemical analysis participated in the German G-EQUAS program and Special Health Quality Control program by the Occupational Safety and Health Research Institute for external quality control. The internal quality control was set using G-EQUAS, Bio-Rad, ClinCheck I and II, and NIST standards in compliance with the quality control guidelines of the NIER. Urinary BPA concentrations were measured by ultra-performance liquid chromatography-mass spectrometry (Xevo TQ-S, Waters, Milford, MA, USA). Values below the detection limit were calculated as the detection limit (0.15 μg/L) divided by √2.

### Outcome

The participants’ height and weight were measured by trained surveyors using a stadiometer and a digital weighing scale of 0.1 cm and 0.1 kg, respectively. Body mass index (BMI) was calculated by dividing weight (kg) by the height squared (m^2^). In the present study, obesity was defined as BMI > 25 kg/m^2^. Obesity is defined as BMI ≥ 30 kg/m^2^ in Western adult populations^26^; however, Western populations generally have lower body fat percentages than East Asians at the same BMI^27^. Therefore, the World Health Organization Western Pacific Region defined obesity in the Asian-Pacific population as BMI > 25 kg/m^2^^2,28^. Since the cutoff value of > 25 kg/m^2^ is widely accepted for defining obesity in the Korean population^29^, we adopted this cutoff to define obesity in our study population.

### Study covariates

The survey included questionnaires on household income, smoking, alcohol consumption, and physical activity. Monthly household income in South Korean Won (KRW) was grouped into three categories (< 1 million KRW, 1–2.99 million KRW, 3–4.99 million KRW, and ≥ 5 million KRW). Smoking status was divided into three groups: never (participants who have never smoked more than 100 cigarettes in their lifetime), former (those who have smoked more than 100 cigarettes in their lifetime but are not currently smoking), and current smoker (those who smoked cigarettes on at least 1 day in the previous 30 days). Alcohol consumption status was categorized into three groups, based on the drinking habits in the past year: non-drinkers (those who did not consume alcohol even once a month), occasional drinkers (those who consumed at least one glass of alcohol every month), and frequent drinkers (those who consumed more than once per week). Regular physical activity was designated as “yes” if the participants performed moderate to intensive physical activity for at least 30 min/day for 5 days/week, or for at least 50 min/day for 3 days/week.

### Adjustment of urinary dilution

We employed a novel method known as CAS to adjust for urinary dilution^19,20^. First, natural log-transformed creatinine was regressed on variables known to affect urinary creatinine levels. In our regression model, we used age, sex, and BMI as covariates. Then, the predicted urinary creatinine (Ucr) was calculated from this model. Then CAS-applied urinary BPA (CAS-BPA) levels were obtained by dividing the measured urinary BPA concentrations by the ratio of the measured to the predicted Ucr, and used to adjust for urinary BPA concentration of each individual.

$$Ln\left( {Predicted \, Ucr} \right) = \beta_{0} + \, \beta_{1} \times \, Age \, \left( {yr} \right) + \beta_{2} \times \, BMI \, \left( {{\text{kg}}/{\text{m}}^{2} } \right) + \beta_{3} \times \, Sex \, \left[ {male = 1,\;female = 2} \right]$$

$${\text{In}}\;{\text{Cycle}}\;2,\upbeta _{0} = 0.306,\;\upbeta _{1} = - \;0.009,\;\upbeta _{2} = 0.015,\;\upbeta _{3} = - \;0.377$$

$${\text{In}}\;{\text{Cycle}}\;3,\;\upbeta _{0} = 0.367,\;\upbeta _{1} = \, - 0.007,\;\upbeta _{2} = 0.015,\upbeta _{3} = - \;0.396$$

$$\text{CAS-applied urinary BPA concentration}=[\text{Urinary BPA concentration}] \times (\text{Predicted Ucr/Ucr}).$$

### Statistical analysis

In accordance with the guidelines of the KoNEHS analysis, statistical analyses were performed by applying the sample weights, stratification, and clustering provided in the original data set. The demographic characteristics and health behaviors of obese participants were analyzed by the Complex samples general linear model (CSGLM) and Complex samples Crosstabs procedure. A comparison of BPA concentrations by demographic and behavioral factors was summarized using the median with the interquartile range due to the asymmetrical distribution. A comparison of the BPA value by demographic and behavioral factors of obesity was analyzed using CSGLM after adjusting for age and sex. CAS-BPA was natural-log-transformed to normalize the asymmetrical distribution (Ln CAS-BPA) for statistical analysis. The association between Ln CAS-BPA and BMI was evaluated using multiple linear regression analysis, adjusted for age, sex, household income, smoking status, alcohol consumption, and physical activity. We conducted multiple logistic regression analysis to estimate odds ratios (ORs) with 95% confidence intervals (CIs) of Ln CAS-BPA for obesity in KoNEHS Cycle 2 and Cycle 3. The pooled OR for KoNEHS Cycle 2 and Cycle 3 was calculated using the Mantel–Haenszel method. For a pooled analysis of individual data from KoNEHS Cycle 2 and Cycle 3, an unweighted analysis was performed because of difficulties in calculating the integrated weights of KoNEHS Cycles 2 and 3. In addition, we performed subgroup analyses with 1:1 propensity score matching (PSM) data to reduce the bias due to confounding factors. We calculated the propensity score to adjust for age, sex, household income, smoking status, alcohol consumption, and physical activity according to obesity status and performed 1:1 nearest neighbor matching. PSM was performed using “MatchIt” package in R^30^.

Changes in the OR of obesity according to BPA levels were determined using a restricted cubic spline plot with four inflection points. The association of ORs, according to the Ln CAS-BPA, was analyzed using a 4-knot restricted cubic spline plot model. Statistical analysis was performed using SPSS version 24.0 (IBM Corp., Armonk, NY, USA) and R version 3.1.0 (the R Foundation for Statistical Computing, Vienna, Austria). For all analyses, *P* values were two-tailed, and a *P* value of < 0.05 was considered significant.

## Results

### Baseline characteristics of the participants

The data of 6,258 participants (2,684 males and 3,574 females) from KoNEHS Cycle 2 and the data of 3,763 participants (1,640 males and 2,123 females) from KoNEHS Cycle 3 were retrieved, and 2,407 and 1,556 individuals were, respectively, classified as obese according to the BMI criteria (Table 1). Participants with obesity tended to be older, male, with lower income, and with higher smoking rates.

**Table 1:** Characteristics of participants in KoNEHS Cycle 2 and Cycle 3 according to obesity status.

Factors	KoNEHS cycle 2			KoNEHS cycle 3		
	Without obesity (N = 3851)	Obesity (N = 2407)	*P* value	Without obesity (N = 2207)	Obesity (N = 1556)	*P* value
**Age, years**	44.8 (43.9–45.6)	48.9 (47.9–49.9)	< 0.001	45.1 (43.9–46.3)	50.0 (48.9–51.0)	< 0.001
**Male, %**	44.7%	56.8%	< 0.001	44.4%	58.2%	< 0.001
**House income, %**			< 0.001			< 0.001
< 1 million KRW	10.4%	13.4%		9.8%	14.8%	
1- 2.99 million KRW	30.2%	34.1%		37.6%	39.8%	
3- 4.99 million KRW	32.7%	30.7%		30.9%	25.7%	
≥ 5 million KRW	26.7%	21.8%		21.7%	19.7%	
**Smoking status, %**			< 0.001			< 0.001
Never	66.3%	56.6%		65.8%	56.0%	
Former	14.1%	18.7%		15.6%	23.1%	
Current	19.6%	24.7%		18.6%	20.9%	
**Alcohol consumption, %**			0.002			0.075
Non-drinker	30.5%	28.3%		27.3%	25.5%	
Occasional drinker	30.0%	26.5%		34.8%	32.0%	
Frequent drinker	39.5%	45.2%		37.9%	42.5%	
**Regular physical activity, %**			0.131			0.039
No	75.9%	73.7%		75.8%	72.0%	
Yes	24.1%	26.3%		24.2%	28.0%	
**BMI, kg/m**^**2**^	22.0 (21.9–22.1)	27.7 (27.6–27.8)	< 0.001	22.2 (22.1–22.3)	27.9 (27.7–28.1)	< 0.001

Obesity status was classified based on BMI.

Values are presented as mean (95% confidence intervals) or percentage (%).

KRW, South Korean Won; BMI, body mass index.

BPA and CAS-BPA levels were significantly higher in participants with obesity in both Cycles (Table 2). In KoNEHS Cycle 3, obese individuals showed significantly higher BPA concentrations in both males and females, but in KoNEHS Cycle 2, higher BPA levels were only statistically significant in females.

**Table 2:** Urinary bisphenol A and CAS-applied urinary bisphenol A according to the participants’ general characteristics and obesity.

Factors	KoNEHS cycle 2				KoNEHS cycle 3			
	Total	Without obesity	Obesity	*P* value*	Total	Without obesity	Obesity	*P* value*
**Urinary bisphenol A (ng/mL)**								
Total	1.12 [0.48;2.53]	1.07 [0.46;2.45]	1.22 [0.52;2.60]	0.010	1.32 [0.52; 2.76]	1.19 [0.46; 2.61]	1.54 [0.62;3.04]	< 0.001
Male	1.17 [0.50;2.62]	1.09 [0.48;2.65]	1.28 [0.55;2.59]	0.400	1.52 [0.58; 3.19]	1.44 [0.51;3.05]	1.70 [0.72;3.31]	0.008
Female	1.07 [0.46;2.39]	1.04 [0.45;2.29]	1.12 [0.51;2.62]	< 0.001	1.14 [0.46; 2.43]	1.09 [0.43;2.26]	1.32 [0.55;2.72]	< 0.001
Age group								
19–29 yr	1.30 [0.59;2.93]	1.28 [0.57;3.05]	1.49 [0.65;2.66]	0.613	1.24 [0.52;2.80]	1.10 [0.45;2.16]	1.58 [0.73;3.06]	0.046
30–39 yr	1.28 [0.56;2.74]	1.18 [0.52;2.60]	1.50 [0.67;2.94]	0.075	1.44 [0.57;2.87]	1.22 [0.47; 2.58]	2.02 [0.84;3.58]	0.002
40–49 yr	1.13 [0.50;2.68]	1.10 [0.49;2.50]	1.20 [0.51;2.76]	0.276	1.37 [0.53;2.93]	1.36 [0.50; 2.89]	1.50 [0.55;2.99]	0.699
50–59 yr	1.11 [0.48;2.49]	1.03 [0.46;2.34]	1.27 [0.58;2.69]	0.089	1.46 [0.52;2.81]	1.30 [0.50;2.55]	1.70 [0.58;3.23]	0.086
60–69 yr	0.94 [0.41;2.02]	0.87 [0.36;2.03]	1.02 [0.47;2.02]	0.204	1.23 [0.49;2.62]	1.09 [0.42;2.59]	1.40 [0.57;2.69]	0.030
≥ 70 yr	0.76 [0.30;1.56]	0.69 [0.29;1.48]	0.81 [0.31;1.69]	0.134	1.11 [0.46;2.23]	1.05 [0.41;2.23]	1.14 [0.56;2.20]	0.433
**CAS-applied urinary bisphenol A (ng/mL)**								
Total	1.08 [0.55;2.13]	1.02 [0.54;2.01]	1.17 [0.57;2.34]	0.006	1.33 [0.62;2.59]	1.24 [0.57;2.41]	1.49 [0.72;2.89]	< 0.001
Male	1.11 [0.55;2.26]	1.05 [0.54;2.18]	1.20 [0.57;2.34]	0.365	1.52 [0.71;3.00]	1.42 [0.65;2.75]	1.59 [0.80;3.24]	0.043
Female	1.03 [0.54;1.99]	0.99 [0.54;1.90]	1.12 [0.57;2.34]	< 0.001	1.15 [0.56;2.28]	1.10 [0.52;2.19]	1.32 [0.65;2.43]	< 0.001
Age group								
19–29 yr	1.28 [0.67;2.48]	1.18 [0.68;2.21]	1.55 [0.60;3.34]	0.349	1.15 [0.56;2.35]	1.03 [0.49;2.19]	1.53 [0.88;2.87]	0.017
30–39 yr	1.17 [0.63;2.33]	1.07 [0.57;2.17]	1.36 [0.73;2.67]	0.005	1.48 [0.72;2.64]	1.35 [0.66;2.40]	1.68 [0.80;3.17]	0.125
40–49 yr	1.11 [0.58;2.24]	1.09 [0.59;2.21]	1.19 [0.56;2.24]	0.939	1.47 [0.61;3.15]	1.44 [0.60;2.83]	1.58 [0.62;3.58]	0.397
50–59 yr	1.10 [0.56;2.27]	0.97 [0.51;2.04]	1.28 [0.65;2.46]	0.050	1.34 [0.68;2.69]	1.35 [0.68;2.63]	1.32 [0.67;3.04]	0.984
60–69 yr	0.93 [0.43;1.72]	0.92 [0.43;1.71]	0.96 [0.43;1.72]	0.637	1.23 [0.62;2.39]	1.07 [0.55;2.28]	1.47 [0.75;2.44]	0.020
≥ 70 yr	0.67 [0.37;1.28]	0.60 [0.37;1.26]	0.73 [0.37;1.34]	0.179	1.06 [0.48;2.22]	0.93 [0.45;2.21]	1.24 [0.51;2.23]	0.092

*General linear model of log-transformed BPA or log-transformed BPA CAS-BPA after adjusting for age and sex.

CAS-BPA, CAS-applied urinary BPA.

Data are presented as median values [interquartile range].

### Association of bisphenol A levels with obesity

**Figure 1:**
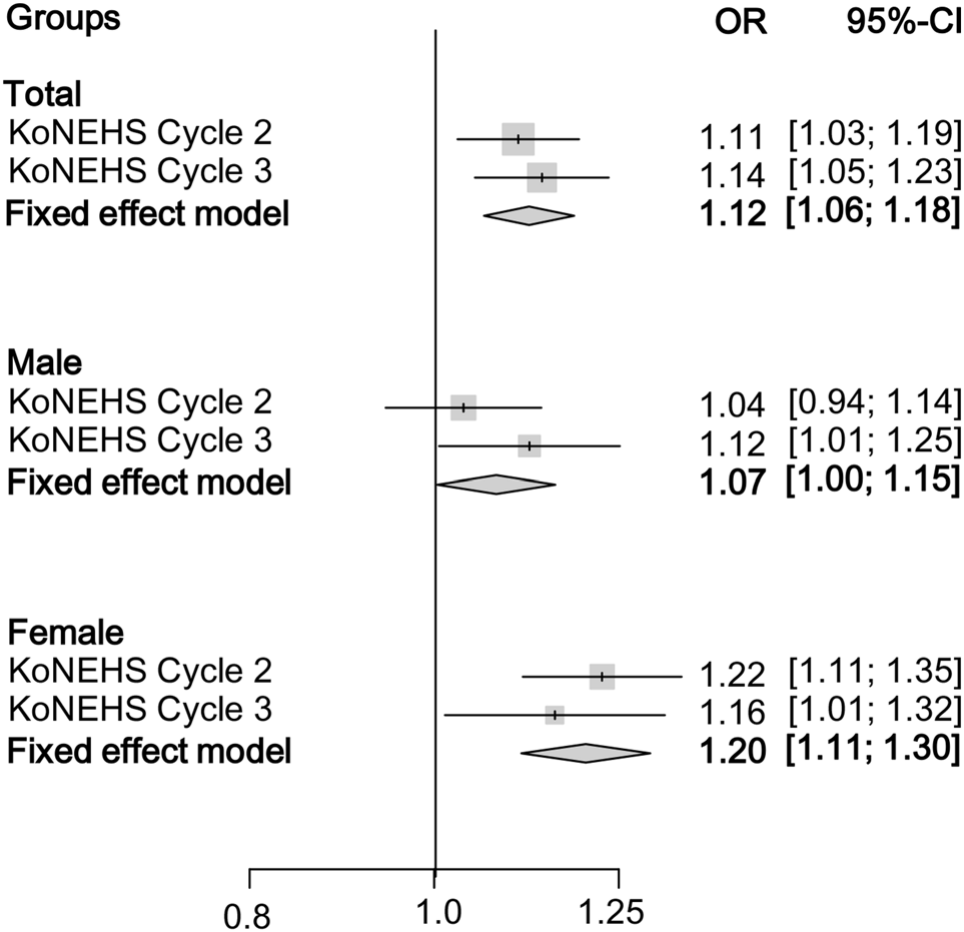
Meta-analysis with data from KoNEHS Cycle 2 and Cycle 3: odds ratios for obesity in males and females. Values were adjusted for age, sex, household income, smoking, alcohol consumption, and physical activity.

In multiple linear regression analysis, Ln CAS-BPA was significantly associated with BMI in KoNEHS Cycle 3 but not in KoNHES Cycle 2 (Table 3). When stratified by sex, there was no association between Ln CAS-BPA levels and BMI in males, but a positive association was noted in females in both Cycles (Table 3). In multiple logistic regression analysis, Ln CAS-BPA was significantly associated with obesity in both KoNEHS Cycle 2 [1.11 (1.03–1.19)] and KoNEHS Cycle 3 [1.14 (1.05–1.23)] (Table 4). When stratified by sex, there was no association between Ln CAS-BPA and obesity in males in KoNEHS Cycle 2 [1.04 (0.94–1.14)], but a positive association was noted in KoNEHS Cycle 3 [1.12 (1.01–1.25)]. In females, significant positive associations were noted in both Cycles [KoNEHS Cycle 2: 1.23 (1.11–1.35), KoNEHS Cycle 3: 1.16 (1.01–1.32)] (Table 4). Similar results were obtained in the meta-analysis of the two data sets (Fig. 1). In the pooled analysis with the data from both Cycles and PSM data, the associations were significant in both males [PSM: 1.07 (1.01–1.14)] and females [PSM: 1.14 (1.08–1.20)] (Table 4). Natural log-transformed BPA with conventional creatinine adjustment (Ln Cr-BPA) showed a significant association with obesity in females, but not in males, in both Cycles (Table 4).

**Table 3:** Association between BMI and urinary bisphenol A.

	KoNEHS Cycle 2		KoNEHS Cycle 3		Pooled data*		PSM data*	
	β (95% CI)	*P* value	β (95% CI)	*P* value	β (95% CI)	*P* value	β (95% CI)	*P* value
**Total**								
Ln Cr-BPA	0.07 (0.01–0.13)	0.028	0.11 (-0.01–0.23)	0.057	0.07 (0.01–0.13)	0.028	0.04 (-0.03–0.11)	0.221
Ln CAS-BPA	0.22 (0.10–0.34)	< 0.001	0.24 (0.13–0.36)	< 0.001	0.21 (0.15–0.27)	< 0.001	0.19 (0.12–0.26)	< 0.001
**Male**								
Ln Cr-BPA	-0.03 (-0.18–0.11)	0.651	0.05 (-0.12–0.21)	0.571	-0.02 (-0.11–0.06)	0.620	-0.02 (-0.11–0.08)	0.694
Ln CAS-BPA	0.10 (-0.05–0.25)	0.170	0.18 (0.02–0.34)	0.024	0.11 (0.03–0.20)	0.009	0.12 (0.03–0.21)	0.026
**Female**								
Ln Cr-BPA	0.19 (0.05–0.33)	0.007	0.17 (0.02–0.33)	0.027	0.15 (0.07–0.23)	< 0.001	0.12 (0.02–0.21)	0.021
Ln CAS-BPA	0.35 (0.21–0.49)	< 0.001	0.29 (0.14–0.44)	< 0.001	0.29 (0.21–0.37)	< 0.001	0.27 (0.17–0.36)	< 0.001

Analysis according to the quartiles of CAS-BPA.

Adjusted for age, sex, household income, smoking, alcohol consumption, and physical activity.

CI, confidence intervals; PSM, propensity score matching; BMI, body mass index; CAS-BPA, CAS-applied urinary BPA.

*Unweighted results.

**Table 4:** Adjusted odds ratios (95% confidence intervals) for obesity according to urinary bisphenol A.

	KoNEHS Cycle 2		KoNEHS Cycle 3		Pooled data*		PSM data*	
	OR	*P* value	OR	*P* value	OR	*P* value	OR	*P* value
**Total**								
Ln Cr-BPA	1.03 (0.96–1.11)	0.464	1.08 (1.01–1.17)	0.034	1.04 (1.01–1.08)	0.023	1.03 (0.99–1.07)	0.113
Ln CAS-BPA	1.11 (1.03–1.19)	0.007	1.14 (1.05–1.23)	0.002	1.12 (1.08–1.16)	< 0.001	1.10 (1.06–1.15)	< 0.001
Analysis according to the quartiles of CAS-BPA								
Quartile 1	Reference		Reference		Reference		Reference	
Quartile 2	0.91 (0.75–1.09)	0.302	1.22 (0.91–1.64)	0.185	1.02 (0.95–1.14)	0.778	1.04 (0.92–1.18)	0.571
Quartile 3	1.22 (1.00–1.48)	0.045	1.26 (0.95–1.68)	0.112	1.21 (1.08–1.36)	0.001	1.20 (1.06–1.37)	0.004
Quartile 4	1.37 (1.09–1.71)	0.007	1.56 (1.20–2.04)	< 0.001	1.41 (1.25–1.58)	< 0.001	1.38 (1.21–1.56)	< 0.001
**Male**								
Ln Cr-BPA	0.97 (0.88–1.07)	0.516	1.05 (0.94–1.16)	0.406	1.01 (0.95–1.06)	0.862	1.01 (0.95–1.07)	0.847
Ln CAS-BPA	1.04 (0.94–1.14)	0.470	1.12 (1.01–1.25)	0.040	1.07 (1.02–1.13)	0.012	1.07 (1.01–1.14)	0.017
Analysis according to the quartiles of CAS-BPA								
Quartile 1	Reference		Reference		Reference		Reference	
Quartile 2	0.78 (0.61–1.00)	0.052	1.34 (0.90–2.00)	0.143	1.00 (0.84–1.19)	0.992	1.02 (0.85–1.23)	0.838
Quartile 3	1.18 (0.88–1.57)	0.273	1.21 (0.81–1.81)	0.343	1.21 (1.01–1.43)	0.035	1.26 (1.05–1.52)	0.013
Quartile 4	1.07 (0.80–1.44)	0.643	1.55 (1.07–2.25)	0.020	1.24 (1.04–1.47)	0.015	1.27 (1.06–1.52)	0.010
**Female**								
Ln Cr-BPA	1.13 (1.03–1.24)	0.014	1.13 (1.02–1.24)	0.024	1.089 (1.034–1.146)	0.001	1.065 (1.007–1.126)	0.027
Ln CAS-BPA	1.23 (1.11–1.35)	< 0.001	1.16 (1.01–1.32)	< 0.001	1.168 (1.110–1.230)	< 0.001	1.138 (1.076–1.203)	< 0.001
Analysis according to the quartiles of CAS-BPA								
Quartile 1	Reference		Reference		Reference		Reference	
Quartile 2	1.12 (0.87–1.43)	0.386	1.07 (0.66–1.73)	0.779	1.06 (0.91–1.24)	0.470	1.07 (0.90–1.26)	0.466
Quartile 3	1.37 (1.05–1.79)	0.019	1.28 (0.86–1.91)	0.223	1.27 (1.09–1.49)	0.003	1.19 (1.00–1.41)	0.049
Quartile 4	1.96 (1.46–2.64)	< 0.001	1.58 (1.07–2.33)	0.023	1.62 (1.38–1.91)	< 0.001	1.52 (1.28–1.81)	< 0.001

Adjusted for age, sex, household income, smoking, alcohol consumption, and physical activity.

PSM, propensity score matching; CAS-BPA, CAS-applied urinary BPA.

*Unweighted results.

**Figure 2:**
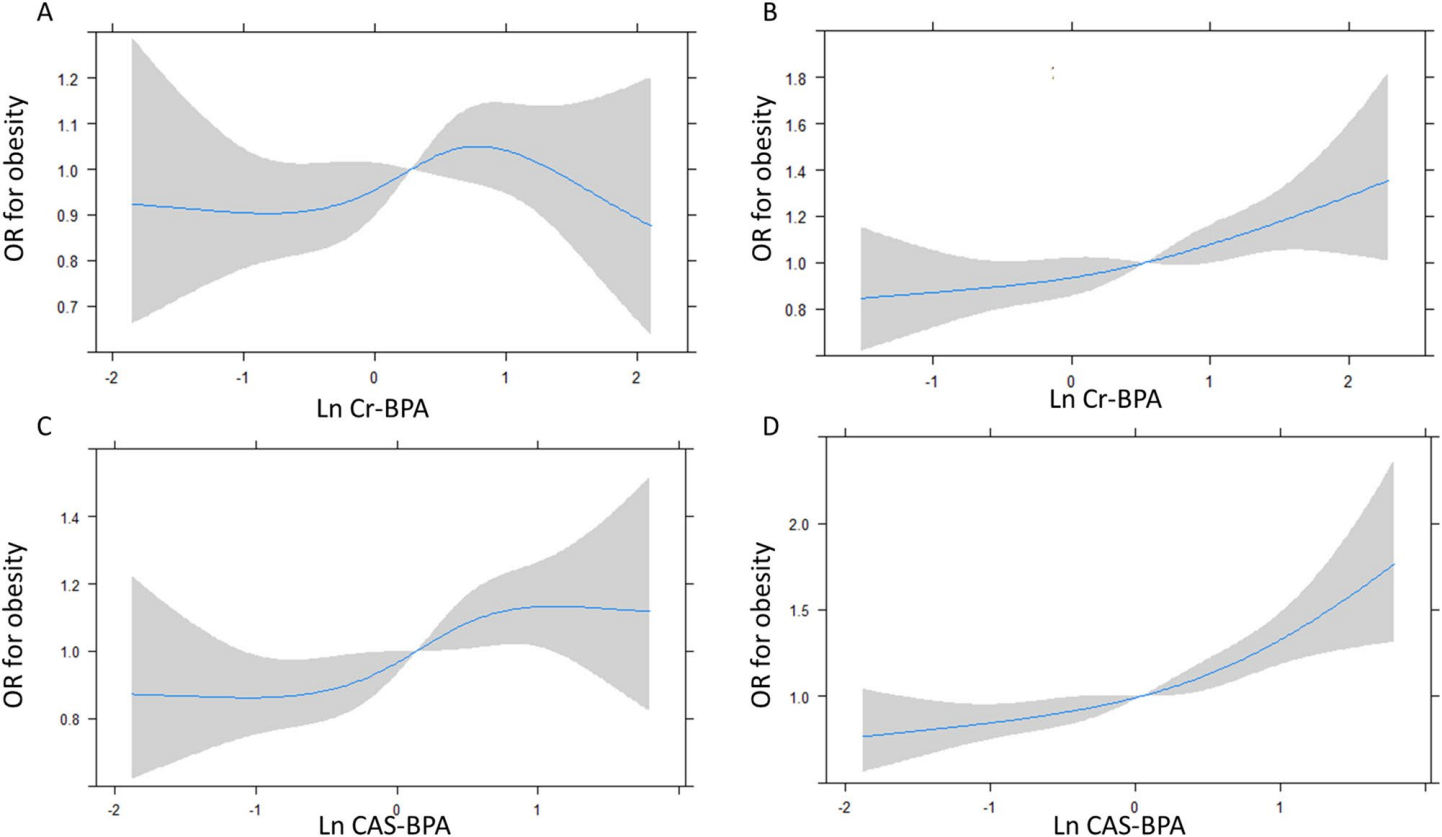
Shape of sex-specific associations between natural log-transformed bisphenol A and obesity risk, which was analyzed using a 4-knot restricted cubic spline plot model with conventional creatinine adjustment (Ln Cr-BPA) [(A) Males, (B) Females] and covariate-adjusted standardization method (Ln CAS- BPA) [(C) Males, (D) Females]. Values were adjusted for age, sex, household income, smoking, alcohol consumption, and physical activity.

Adjusted ORs (95% CIs) for obesity, according to the quartiles of CAS-BPA, are shown in Table 4. In males, the mean value of BMI was 24.5 kg/m^2^ (24.3–24.7) in quartile 1 (Q1), 24.5 kg/m^2^ (24.3–24.7) in quartile 2 (Q2), 24.8 kg/m^2^ (24.6–25.0) in quartile 3 (Q3), and 24.9 kg/m^2^ (24.8–25.2) in quartile 4 (Q4) (p-trend < 0.001). No significant association was noted in KoNEHS Cycle 2 [Q4: 1.07 (0.80–1.44)], but significantly higher ORs were observed in Q4 [1.55 (1.07–2.25)] compared with those of Q1 in KoNEHS Cycle 3. In females, the mean BMI was 24.0 kg/m^2^ (23.9–24.2) in Q1, 24.0 kg/m^2^ (23.8–24.1) in Q2, 24.1 kg/m^2^ (24.0–24.3) in Q3, and 24.6 kg/m^2^ (24.4–24.8) in Q4 (p-trend < 0.001). ORs were significantly elevated according to the increase in CAS-BPA in a dose-dependent manner in both KoNEHS Cycle 2 (p-trend < 0.001) and KoNEHS Cycle 3 (p-trend < 0.001) (Table 4). Pooled analysis with data from KoNEHS Cycle 2 and Cycle 3 and PSM data showed significantly higher ORs for obesity in CAS-BPA Q3 and Q4 compared with that in Q1 in both males and females (Table 4). The association of ORs for obesity with Ln CAS-BPA in a 4-knot restricted cubic spline plot is presented in Fig. 2. When the conventional creatinine adjustment method was applied, a positive association between Ln Cr-BPA levels and the ORs for obesity was observed in females (p < 0.01), but not in males (p = 0.329). However, Ln CAS-BPA was positively associated with ORs for obesity in both sexes, which were more prominent in females (p < 0.01) than in males (p = 0.01).

## Discussion

In this study, significant positive associations were observed between urinary BPA and the risk of obesity using the KoNEHS (2012–2017) data. To the best of our knowledge, this is the largest epidemiological study conducted on this topic and including more than 10,000 participants. Additionally, this is the first study that used novel CAS-applied urinary BPA levels to circumvent potential collider issues in the association between BPA exposure and obesity. The use of urinary creatinine has been one of the most frequently used methods for correction of urinary dilution. However, urinary creatinine is mainly produced from skeletal muscle; therefore, obese individuals with more skeletal muscle compared with lean individuals would excrete more creatinine in the urine^31^. Moreover, urinary creatinine levels are also affected by age and sex^17^. Therefore, using creatinine as a separate covariate to adjust for urinary dilution in obesity research could cause collider stratification bias, offsetting the association with obesity^18^. Recent studies suggested that the collider issues could be solved to some extent by including the fitted urinary creatinine concentration in the urine dilution adjustment factor, which is called CAS^19,20^. Our observation is compatible with these previous reports. While the conventional creatinine adjustment method showed a null association between urinary BPA and obesity in the male population of the KoNHES Cycle 2 and 3, this new approach showed a positive association between urinary BPA and obesity in the same population.

Our finding is consistent with most of the previous epidemiologic studies conducted among adults from different ethnicities. In a previous study from the US NHANES 2003–2006, participants in the upper BPA quartiles were more likely to be obese compared with those in the lowest BPA quartile^10^. A subsequent study of participants from NHANES 2003–2008 identified positive associations between urinary BPA and ORs for obesity in both males and females^12^. Moreover, other studies from Chinese and Canadian populations reported increasing ORs for obesity across increasing BPA quartiles^11,13^.

Detailed biological mechanisms linking BPA exposure and obesity are still unknown, but a variety of explanations have been proposed. In vitro studies have shown that BPA stimulates adipocyte differentiation and accelerates triacylglycerol deposition in fibroblasts^32^. BPA also activates the peroxisome proliferator‐activated receptor γ gene, which is crucial in the regulation of adipogenesis and energy metabolism^33^. In addition, BPA enhanced adipogenesis in adipose stromal cells, possibly through an estrogen receptor-mediated pathway^34,35^.

In this study, we found a sex-related difference in positive associations between BPA and obesity among a nationally representative adult population of a large size, which is linear in females and non-linear in males. There have been a few human studies showing the sex-specific difference in the impact of BPA on obesity. A previous study from NHANES 2003–2008 identified that females showed a positive association between urinary BPA and obesity in a concentration-dependent manner. On the other hand, in males, the relationship was positive but not linear, which is consistent with our finding^12^. Likewise, a few pediatric studies from the US and China reported stronger associations between BPA and adiposity in girls than in boys^36,37^. However, other studies showed stronger positive associations in boys than in girls^38,39^, or no sex-related difference in the associations^40^. We speculated that the sex-specific associations in our finding might be attributed to the sex-specific effects of BPA. Adipose tissue constitutes one of the main target tissues of BPA toxicity but may also be a reservoir for BPA^41^. Therefore, higher adipose tissue reserves in females might render females more susceptible to BPA toxicity compare to males. Furthermore, the anti-androgenic effect of BPA might interfere with the muscle-building impact of testosterone in males^42^. Reduced muscle mass in males with higher BPA exposure might result in a non-linear BPA-BMI association, which is supported by several epidemiologic studies showing the inverse associations of BPA and serum testosterone in males^43–45^. These hypotheses might partly explain the stronger associations between BPA exposure and obesity risk in females compared with males in this study. Sex-specific effects of BPA on obesity should be further investigated in human epidemiologic studies that involve analyses of body composition and sex hormone levels.

In this study, we showed that the conventional creatinine adjustment method could mask the associations between urinary BPA levels and BMI-based obesity status, especially in males. Since BMI reflects both fat mass and muscle mass at the same time, the relatively higher muscular mass in males might increase the collider bias when using creatinine as a covariate in the BMI-based outcome model. By adopting CAS-BPA levels and minimizing the potential bias, we could unveil the positive associations between BPA exposure and obesity risk not only in females but also in males.

Our study had some limitations. First, the sources of BPA exposure, including occupational exposure, types of food containers, frequency of plastic products or canned foods, and dietary calorie intakes, were not assessed. Waist circumference and body composition analyses, which reflect adiposity more precisely, were not measured in this study. As a result of the cross-sectional nature of this study, the observed association here could not infer a causal relationship with obesity. Lastly, due to a short biological half-life of BPA, single urine BPA concentrations do not reflect prolonged exposure^46^. Nonetheless, to the best of our knowledge, this is the first study to determine the positive associations of BPA and obesity using a novel CAS method in the largest scale national biomonitoring data and to show the sex-specific effects on obesity risk in relation to urinary BPA concentrations. Several hypotheses derived for sex-related difference in the association would facilitate both epidemiologic and experimental studies which will improve our understanding of the involvement of BPA in the development of obesity.

## Conclusion

This study used a novel CAS method to account for urinary dilution and showed sex-specific positive associations between BPA exposure and obesity. While a non-linear positive association was observed in males, a positive linear association was observed in females. Further experimental and prospective epidemiological studies are needed to confirm these findings.

## Supplementary information

Supplementary Figures.

## Abbreviations

BMI: Body mass index
BPA: Bisphenol A
KoNEHS: Korean National Environmental Health Survey
