# Supplementary material for: Urinary bisphenol A concentrations and the risk of obesity in Korean adults
Source: Sci Rep. 2021 Jan 15;11:1603. doi: 10.1038/s41598-021-80980-8 (PMC7811011; doi:10.1038/s41598-021-80980-8)
Supplement: Supplementary file 1 — Supplementary Figures. [file 41598_2021_80980_MOESM1_ESM.docx]

**Urinary bisphenol A concentrations and the risk of obesity in Korean adults**

Shinje Moon^a^, Moon Young Seo^b^, Kyungho Choi^c^, Yoon-seok Chang^d^, Shin-Hye Kim^b,^*, Mi Jung Park^b,^*

^a^Department of Internal Medicine, Kangnam Sacred Heart Hospital, Hallym University Medical Center, Hallym University College of Medicine, Seoul 07441, Republic of Korea

^b^Department of Pediatrics, Sanggye Paik Hospital, Inje University College of Medicine, Seoul 01757, Republic of Korea

^c^Department of Environmental Health Sciences, Seoul National University, Seoul 08826, Republic of Korea

^d^Division of Environmental Science and Engineering, Pohang University of Science and Technology (Postech), Pohang, 37673, Republic of Korea

**Corresponding authors:**

*Mi Jung Park

Department of Pediatrics, Sanggye Paik Hospital, Inje University College of Medicine, 1342, Dongilro, Nowon-gu, Seoul 01757, Korea

Tel: +8229504812, Fax: +8229501246, E-mail address*:* PMJ@paik.ac.kr

*Shin-Hye Kim, MD

Department of Pediatrics, Inje University Sanggye Paik Hospital, Inje University College of Medicine, 1342 Dongil-ro, Nowon-gu, Seoul 01757, Korea

Tel: +82-2-950-4812, Fax: +82-2-950-1246, E-mail: S2635@paik.ac.kr

**Supplementary Figure S1.** Flowchart showing the final selection process

**
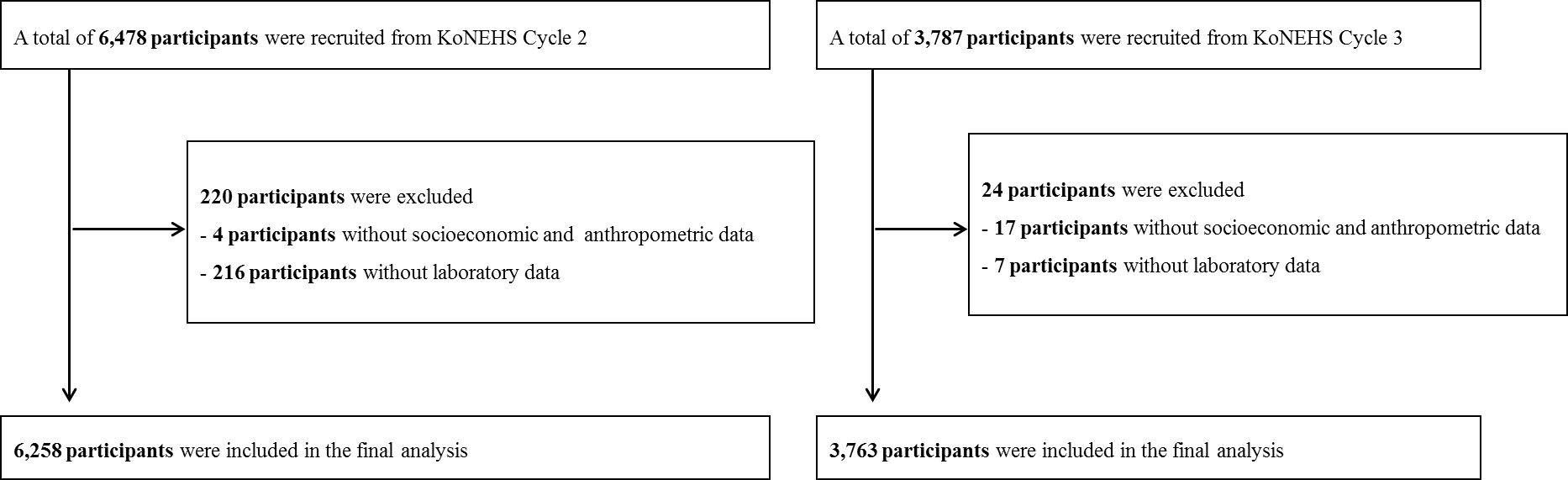
**
